# δ PREDICTS GENERAL PSYCHOPATHOLOGY

**DOI:** 10.1093/geroni/igz038.2378

**Published:** 2019-11-08

**Authors:** Donald R Royall, Raymond F Palmer

**Affiliations:** 1 Departments of Psychiatry, Medicine and Family & Community Medicine, The University of Texas Health Science Center at San Antonio, San Antonio, Texas, United States; 2 Department of Family & Community Medicine, The University of Texas Health Science Center at San Antonio, San Antonio, Texas, United States

## Abstract

“δ” is a transdiagnostic omnibus dementia severity measure derived from general intelligence (i.e., Spearman’s “g”). It can be estimated in any cognitive battery that contains a measure of Instrumental Activities of Daily Living (IADL). As dementia’s essential cognitive impairment, δ may also explain the appearance of Behavioral and Psychological Symptoms of Dementia (BPSD). Alternatively, the latter may be associated with orthogonal domain-specific cognitive impairments, unrelated to δ and therefore to dementia. The δ homolog ”dDx” was tested as a predictor of one year prospective BPSD among n = 723 participants in the Texas Alzheimer’s Research and Care Consortium (TARCC). Twelve Neuropsychiatric Inventory (NPI-Q)-rated BPSD were themselves submitted to confirmatory factor analysis resulting in a well-fit bifactor model rating general psychopathology (p), positive (+) and negative (-) symptoms. dDx and orthogonal cognitive factors rating memory (MEM) and executive function (EF) were regressed onto prospective p, (+) and (-). dDx was strongly associated with p (r = -0.59, p <0.001). MEM was associated only with (+) (r = 0.14, p <0.001). EF was associated only with (-) (r = -0.23, p <0.001). This is the first demonstration of p in the geriatric literature. p’s strong association with δ suggests that general psychopathology arises from dementia itself. In contrast, (+) and (-) symptoms may arise from regional insults, e.g., to temporo-limbic and frontal circuits (respectively). No such regional pathology is likely to impact δ or p, as they are “indifferent” to their indicators and must derive from global Central Nervous System (CNS) features.

